# 48-Week Outcome after Cessation of Nucleos(t)ide Analogue Treatment in Chronic Hepatitis B Patient and the Associated Factors with Relapse

**DOI:** 10.1155/2018/1817680

**Published:** 2018-05-10

**Authors:** Wen-xiong Xu, Qian Zhang, Xiang Zhu, Chao-shuang Lin, You-ming Chen, Hong Deng, Yong-yu Mei, Zhi-xin Zhao, Dong-ying Xie, Zhi-liang Gao, Chan Xie, Liang Peng

**Affiliations:** ^1^Department of Infectious Diseases, Third Affiliated Hospital of Sun Yat-sen University, Guangzhou, Guangdong 510630, China; ^2^Guangdong Key Laboratory of Liver Disease Research, Third Affiliated Hospital of Sun Yat-sen University, Guangzhou, Guangdong 510630, China; ^3^Department of Infectious Diseases, Union Hospital, Tongji Medical College, Huazhong University of Science and Technology, Wuhan, Hubei 430022, China

## Abstract

**Background and Aims:**

We aimed to ascertain the feasibility and safety of NA cessation, the status of patients after cessation, and the predictive factors for relapse and subsequent retreatment.

**Methods:**

A total of 92 patients were enrolled in this prospective study. Patients were monitored every month for the first 3 months after cessation and every 3 months thereafter.

**Results:**

Sixty-two patients finished 48 weeks of follow-up. None died or developed liver failure, cirrhosis, or HCC. The 62 patients could be divided into 4 categories according to the 48-week clinical development of relapse. Virologic relapses occurred in 39 (62.9%) patients, with 72.7% occurring in the first 24 weeks in origin HBeAg positive patients and 82.4% in the first 12 weeks in origin HBeAg negative patients. Age (OR = 1.06, 95% CI = 1.02–1.10; *p* = 0.003), the HBsAg level (OR = 2.21, 95% CI = 1.47–3.32; *p* < 0.001), and positive origin HBeAg status (OR = 0.32, 95% CI = 0.14–0.74; *p* = 0.008) were predictive factors to virologic relapse. HBV DNA level (OR = 1.34, 95% CI = 1.13–1.58; *p* < 0.001) was predictive factor to retreatment.

**Conclusions:**

NA cessation is safe under supervision. Age, HBsAg level, and origin HBeAg status can be predictive factors for virologic relapse. The study was submitted to ClinicalTrials.gov Protocol Registration and Results System with the assigned NCT ID NCT02883647.

## 1. Introduction

Chronic hepatitis B virus (HBV) infection is a major health problem, as approximately 240 million people are infected worldwide [1]. Nucleos(t)ide analogues (NA) have been developed to control HBV [2], and they can strongly inhibit reverse transcriptase to suppress the HBV DNA levels but cannot erase covalently closed circular DNA (cccDNA), as it integrates into the genome in the nuclei of the host hepatocytes [3]. Therefore, most patients require long-term NA therapy, even though HBV DNA levels are suppressed to undetectable levels in a short amount of time.

Long-term NA therapy can control chronic hepatitis B, but several problems remain. The first is the economical burden, especially in developing countries where health care and life insurance are poor. The second are poor adherence and retention in care of the patients, resulting from this high cost. The third is the side effect of the drugs, including renal and bone toxicity. The fourth are viral mutant and drug resistance which are further hindrances to drug efficacy. Finite NA therapy has the advantages of solving the above problems. How to make finite NA therapy is what physicians and patients concern.

Guidelines of prevention and treatment of chronic hepatitis B (CHB) from China [4], Asian Pacific Association for the Study of the Liver (APASL) [5], European Association for the Study of the Liver (EASL) [6], American Association for the Study of Liver Diseases (AASLD) [7], and World Health Organization (WHO) [8] all make suggestions on NA cessation, including parameters like NA treatment duration and consolidation treatment duration. Although physicians follow these suggestions, recent studies still indicate high virologic relapse rates ranging from 40% to 95% in the first year after NA cessation [9–12]. Some authors in these studies presented predictive factors for relapse, such as age, consolidation treatment duration, NA treatment duration, and baseline HBV DNA level. But some authors had the opposite opinion. There are, as yet, difficulties of how to stop NA therapy without relapse. Otherwise, NA cessation without supervision may result in an unpredictable worsening of disease and the possible development of cirrhosis, fulminant hepatitis, or acute-on-chronic liver failure (ACLF). Safety of NA therapy cessation and NA retreatment efficacy after relapse are very important issues that should be found out. In this study, we aimed to determine the feasibility and safety of NA cessation, the therapeutic effect of retreatment in relapsed patients, the features of patients after cessation, and the predictive factors for relapse and retreatment.

## 2. Materials and Methods

### 2.1. Study Population

This prospective study recruited 92 Chinese HAN outpatients with chronic hepatitis B in Third Affiliated Hospital of Sun Yat-sen University from December 2013 to January 2017. All patients were treated with nucleos(t)ide analogues, such as entecavir (ETV), telbivudine (LDT), lamivudine (LAM), adefovir dipivoxil (ADV), or a combination of LAM and ADV. Informed consent from the patients was obtained.

### 2.2. Inclusion and Exclusion Criteria

*Inclusion Criteria*. They include the following: (1) CHB patients (hepatitis B surface antigen [HBsAg] and HBV DNA positive for at least 6 months [5]) receiving NA therapy; (2) age from 18 to 65 years; (3) patients who had been treated with NA for at least 2 years with undetectable HBV DNA levels on at least 3 separate occasions, 6 months apart, before the cessation of treatment (otherwise, hepatitis B e antigen (HBeAg) seroconversion was required and maintained for at least one year in origin HBeAg positive patients); (4) HBV DNA level that were “not detected” or “<20 IU/mL,” as determined by Roche COBAS detection before cessation.

*Exclusion Criteria*. They include the following: (1) patients with liver cirrhosis, hepatocellular carcinoma (HCC), or other malignancies; (2) patients with other factors causing liver disease; (3) pregnant or lactating women; (4) patients with concomitant HIV infection or congenital immune deficiency diseases; (5) patients with diabetes or autoimmune diseases; (6) patients with important organ dysfunctions or serious complications (e.g., infection, ascites, hepatic encephalopathy, hepatorenal syndrome, or gastrointestinal bleeding).

### 2.3. Follow-Up Evaluation

All patients were monitored every month for the first 3 months after cessation and every 3 months thereafter. Symptoms (e.g., fatigue, poor appetite, and jaundice), occurrence of liver cirrhosis or HCC, and mortality were all recorded for the study. Blood cells (white blood cells, red blood cells, hemoglobin, and platelets), biochemical parameters (serum aspartate transaminase [AST], alanine transaminase [ALT], total bilirubin, blood urea nitrogen [BUN], and creatinine), virologic parameters (quantitative HBsAg, HBeAg, HBeAb, and HBV DNA), T lymphocytes (CD4 positive T lymphocytes [CD4^+^T], CD8 positive T lymphocytes [CD8^+^T], Type 1 helper T lymphocytes [Th1], and Type 2 helper T lymphocytes [Th2]), and ultrasound results were assessed at every visit.

Routine automated techniques were used for all biochemical tests at our clinical laboratories. Serum HBsAg levels were measured using Roche Elecsys HBsAg II quant assay (range 0.05–52000 IU/mL, Roche Diagnostics, Mannheim, Germany). Serum HBeAg and HBeAb were assayed using the EIA kit (Abbott Diagnostics, North Chicago, IL). Serum HBV DNA levels were measured with real-time PCR using the COBAS AmpliPrep/COBAS TaqMan HBV Test, version 2.0 (detection limit: 20 IU/mL, Roche Molecular Systems, Inc., Branchburg, NJ, USA). T lymphocytes were measured using flow cytometry analysis with a BD Accuri C6 flow cytometer according to the manufacturer's instructions.

### 2.4. Definition and Management of Relapse

According to guideline of prevention and treatment of chronic hepatitis B from APASL [5], virologic relapse was defined as HBV DNA > 2000 IU/mL, while clinical relapse was defined as HBV DNA > 2000 IU/mL and ALT > 2 times upper limit of normal (ULN).

*Patients Management*. (1) Patients with nonrelapse would go on for the next visit. (2) Patients with virologic relapse would go on for the next visit if ALT ≤ 2 times ULN. (3) Patients with clinical relapse would go on for the next visit if they have no symptoms and ALT ≤ 5 times ULN. (4) Patients with clinical relapse would be retreated with NA if they have symptoms and ALT ≤ 5 times ULN. (5) Patients with clinical relapse would be retreated with NA if ALT > 5 times ULN. Patients were monitored every month for the first 3 months after retreatment with NA and every 3 months thereafter.

### 2.5. Ethical Approval

Ethical approval was provided by the Ethics Committee of Medical Clinical Trials, the Third Affiliated Hospital of Sun Yat-sen University (March 20, 2015).

### 2.6. Statistical Analysis

Continuous data were indicated with mean ± SD while categorical data were reported as number and percentage (%). Spearman correlation coefficient was used to investigate the correlation among HBsAg, HBV DNA, and T lymphocytes. Nonparametric tests including Mann–Whitney *U* test and Wilcoxon signed-rank test were used to compare means between groups for data normality was not assumed.

The outcome variables included the occurrences of virologic relapse or retreatment at baseline (month 0) and 1, 2, 3, 6, and 12 months after cessation. Associations between independent variables and outcome variables were analyzed using univariate/multivariate generalized estimating equation (GEE) and binary logistic regression models. An independent working correlation matrix was adopted for the repeated measure data. ROC analysis was further used to assess the diagnostic effectiveness of independent variables which were found associated with outcome. The statistical significance level for all the tests was set at a *p* value < 0.05. Statistical analyses were performed using IBM SPSS Version 20 (SPSS Statistics V20, IBM Corporation, Somers, New York).

## 3. Results

### 3.1. Baseline Characteristics

**Figure 1:**
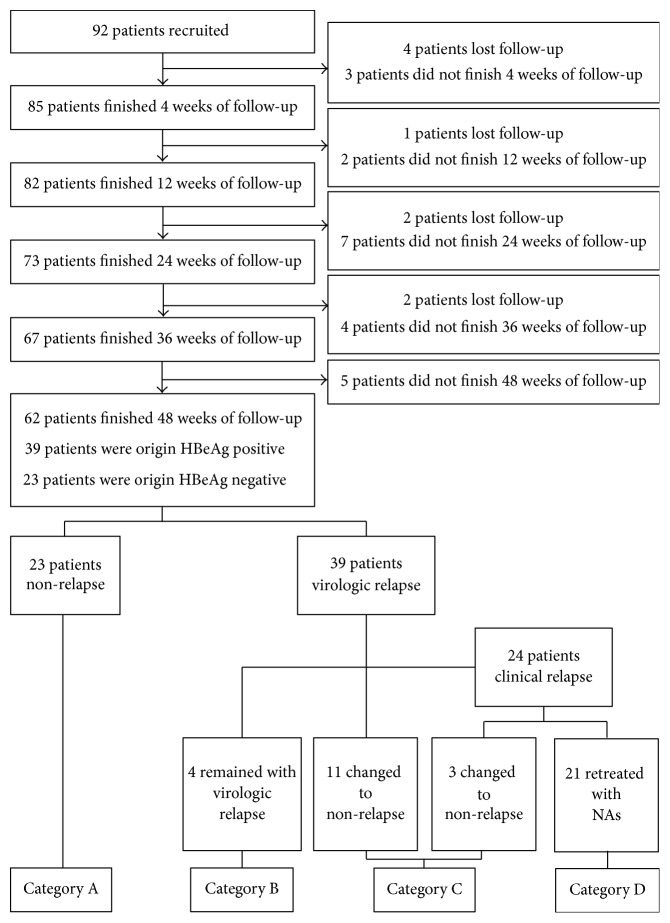
Flow chart of patient recruitment and clinical development.

A total of 92 patients treated with NA were recruited in this study. Nine of the patients were lost to follow-up, and 62 finished the 48 weeks of follow-up. Thirty-nine of the 62 patients were origin HBeAg positive before NA treatment; they gained HBeAg seroconversion before NA cessation. Twenty-three patients were origin HBeAg negative before NA treatment. Nineteen patients were treated with ETV for 4.55 ± 1.93 years before cessation, 23 patients were treated with LDT for 3.35 ± 1.34 years, 5 patients were treated with LAM for 5.02 ± 2.46 years, 7 patients were treated with ADV for 7.51 ± 2.65 years, and 8 patients were treated with combination of LAM and ADV for 4.94 ± 2.95 years. The flow of patient recruiting and clinical development was indicated in Figure 1. The baseline characteristics of the 62 patients were shown in Table 1.

### 3.2. Safety of NA Cessation

In the course of the 48 weeks of follow-up, none of the 62 patients died or developed liver failure, cirrhosis, or hepatocellular carcinoma. Twenty-one (33.9%) patients were retreated with NA (origin NA or ETV or TDF) and regained normal ALT and undetectable HBV DNA within 24 weeks.

### 3.3. Four Categories in Follow-Up

**Figure 2:**
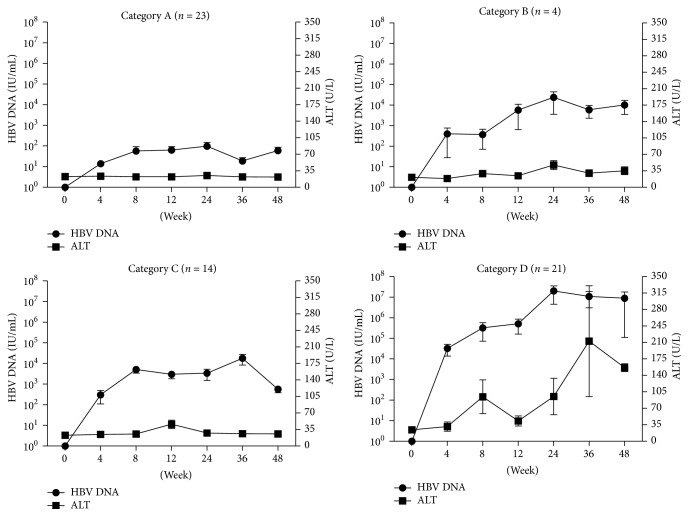
Change of levels of ALT and HBV DNA in four categories from 0 to 48 weeks.

According to the development of patient's levels of HBV DNA and ALT across time (from 0 to 48 weeks) and features of relapse in the follow-up, the 62 patients could be divided into 4 categories (Figures 1 and 2): Category A, consisting of 23 patients in the nonrelapse group, for whom ALT remained normal and HBV DNA levels were not higher than 2000 IU/mL during the follow-up; Category B, consisting of 4 patients in the virologic relapse group, for whom ALT levels were not higher than 2 ULN and HBV DNA remained above 2000 IU/mL during the follow-up; Category C, consisting of 14 patients in the virologic relapse group, for whom HBV DNA levels decreased into no higher than 2000 IU/mL automatically without antiviral treatment during the follow-up, changing patient status to nonrelapse; Category D, consisting of 21 patients with high ALT levels and HBV DNA levels which were sufficiently elevated during the follow-up to necessitate retreatment with NA, in order to avoid fulminant hepatitis or cirrhosis.

### 3.4. Cumulative Relapse and Retreatment Rates

**Figure 3:**
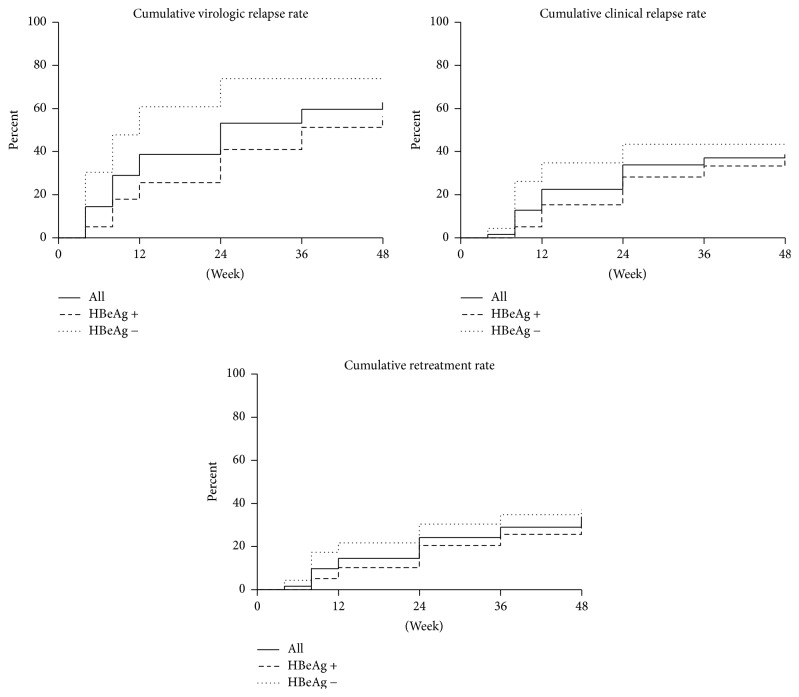
Forty-eight-week cumulative rates of virologic relapse, clinical relapse, and retreatment.

For all 62 patients who finished 48-week follow-up, the 48-week cumulative virologic relapse, clinical relapse, and retreatment rate were 62.9%, 38.7%, and 33.9%, respectively. For the 39 origin HBeAg positive patients, the 48-week cumulative virologic relapse, clinical relapse, and retreatment rate were 56.4%, 35.9%, and 30.8%, respectively. Sixteen of 22 (72.7%) virologic relapses and 11 of 14 (78.6%) clinical relapses occurred in the first 24 weeks in origin HBeAg positive patients. For the 23 origin HBeAg negative patients, the 48-week cumulative virologic relapse, clinical relapse, and retreatment rate were 73.9%, 43.5%, and 39.1%, respectively. Fourteen of 17 (82.4%) virologic relapses and 6 of 10 (60%) clinical relapses occurred in the first 12 weeks in origin HBeAg negative patients. The results were shown in Figure 3.

### 3.5. CD4^+^T, CD8^+^T, Th1, and Th2

**Figure 4:**
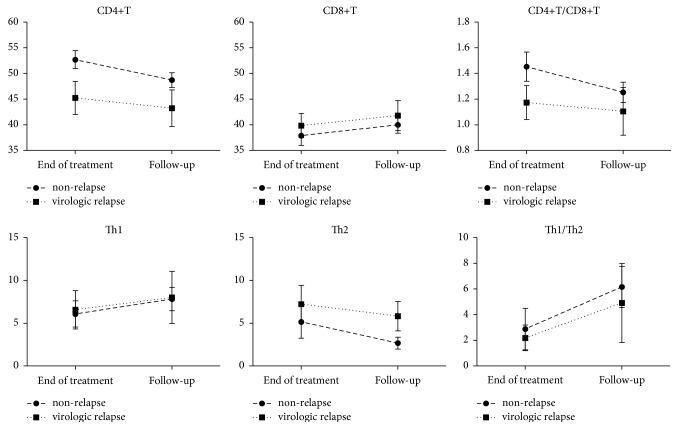
Changes in the ratios of CD4^+^T, CD8^+^T, Th1, and Th2 in nonrelapse and virologic relapse patients.

To further investigate the association among HBsAg, HBV DNA, and flow cytometry results, correlation analyses were used. A negative correlation was found between HBsAg and CD4^+^T (*r* = −0.37, *p* < 0.001), a positive correlation was found between HBsAg and CD8^+^T (*r* = 0.37, *p* < 0.001), and no correlations at all were found between HBsAg and Th1 (*p* = 0.832) or Th2 (*p* = 0.887). A negative correlation was found between HBV DNA and CD4^+^T (*r* = −0.44, *p* < 0.001), and no correlations at all were found between HBV DNA and CD8^+^T (*p* = 0.114) or Th1 (*p* = 0.243) or Th2 (*p* = 0.703). The changes in these ratios over the course of follow-up in both nonrelapse and virologic relapse patients were shown in Figure 4. No statistical significance was found between any of the groups (all *p* > 0.05).

### 3.6. Predictive Factors for Virologic Relapse and Retreatment

Univariate and multivariate logistic regression under GEE models were used to investigate the possibly predictive factor for virologic relapse or retreatment. Independent variables which were not significant in univariate results would still be modeled in multivariate model as adjustment of covariates. Only the variables which reached significance in both univariate and multivariate models would be recognized as possibly predictive factor to virologic relapse or retreatment.

As shown in Table 2, age and the level of HBsAg were significant risk factors. As age and the level of HBsAg are increasing, the more likely patients would occur with virologic relapse. The estimated ORs (odds ratio) of age and the level of HBsAg were 1.06 (95% CI = 1.02–1.10; *p* = 0.003) and 2.21 (95% CI = 1.47–3.32; *p* < 0.001), respectively. On the contrary, positive status of origin HBeAg before NA treatment was a protective factor. The estimated OR was 0.32 (95% CI = 0.14–0.74; *p* = 0.008) which means lower risk to occur with virologic relapse.

The results of retreatment were indicated in Table 3. The level HBV DNA was found to be the only variable which was significant in both univariate and multivariate models. The estimated OR of HBV DNA was 1.34 (95% CI = 1.13–1.58; *p* < 0.001) which means every unit increased would increase 1.34 times of odds of needing retreatment.

**Figure 5:**
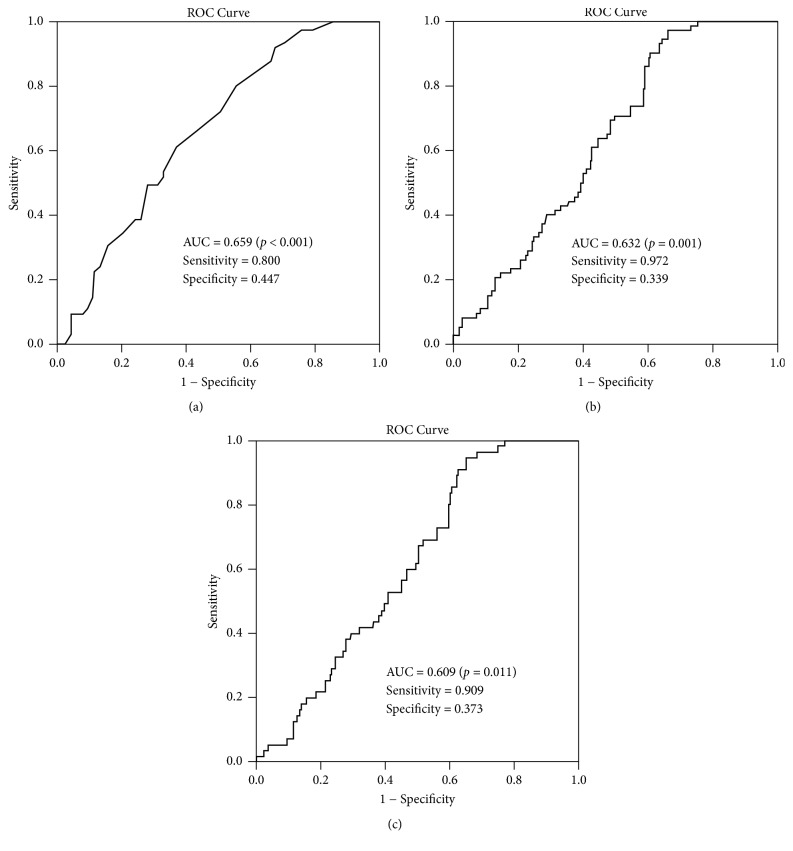
ROC curve and parameters of associated factors, including age to virologic relapse (a), the level of HBsAg to virologic relapse (b), and the level of HBsAg to clinical relapse (c).

ROC analysis was further used to investigate the diagnostic effectiveness of these associated factors. As indicated in Figure 5, the results of age to virologic relapse (Figure 5(a)), the level of HBsAg to virologic relapse (Figure 5(b)), and the level of HBsAg to clinical relapse (Figure 5(c)) all showed significant diagnostic effectiveness (all *p* < 0.05). The optimal cutoffs chosen by Youden's index of age to virologic relapse, the level of HBsAg to virologic relapse, and the level of HBsAg to clinical relapse were 32.5 years, 2.05 log⁡10(IU/mL), and 2.30 log⁡10(IU/mL), respectively. All these three ROC results demonstrated good sensitivity (over 0.8) but lower specificity (lower than 0.5); however these results still indicated that age and HBsAg were potential factors in predicting relapse.

## 4. Discussion

In this study, under supervision, none of the patients in our study died or developed cirrhosis, liver failure, or hepatocellular carcinoma. The 48-week cumulative virologic relapse rates in origin HBeAg positive and negative CHB patients were 56.4% and 73.9%, respectively, which were similar results to a study [13] by Lee et al. and a systematic review [14] by Chang et al., although we strictly followed the 2012 APASL guidelines [5]. Twenty-one (39.1%) patients required retreatment with NAs. Fortunately, the virologic relapse was controlled by retreatment within 24 weeks.

Increased attention should be paid during the first 24 weeks of the follow-up after NA cessation in origin HBeAg positive patients, since 16 of 22 (72.7%) virologic relapses and 11 of 14 (78.6%) clinical relapses occurred in these first 24 weeks. Similarly, the first 12 weeks of follow-up after NA cessation also require increased vigilance in origin HBeAg negative patients, since 14 of 17 (82.4%) virologic relapses and 6 of 10 (60%) clinical relapses occurred in these first 12 weeks.

The 62 patients could be divided into four categories in this study. We designated Category A as “immune control,” since no relapse occurred in the category. We designated Category B as “immune retolerance,” since we assumed that the immune status of these patients is similar to “immune tolerance” in childhood during chronic HBV infection with a high load of HBV replication and normal ALT levels. We designated Category C as “immune recontrol,” since we assumed that host immunity blocked HBV replication without help from the NA therapy. We designated Category D as “immune reactivation,” since we assumed these patients were similar to naïve patients receiving NA therapy with elevated ALT and HBV DNA levels. The four different immune statuses may relate to the function of HBV-specific T cells, because the antiviral drug-induced attenuation of viremia provides a window for the reconstitution of the HBV-specific immune response [15]. Further study of HBV-specific T cell response should be conducted, in order to ascertain the differences between these four categories.

In this study, we found a negative correlation between HBsAg and CD4^+^T, a positive correlation between HBsAg and CD8^+^T, and a negative correlation between HBV DNA and CD4^+^T. In patients with chronic HBV infection, cytotoxic T cell response is weak [16], especially for virus-specific T cell response [17]. CD8^+^T activity is inhibited by high levels of HBV DNA, microRNA-146a, and immunosuppressive cytokines such as interleukin-10 [18, 19]. Therefore, removal of viral antigens, allowing T cells to rest, is important for functional reconstitution of T cell immune response [20]. The use of NA to reduce viremia helps functional recovery of HBV-specific immune response [15].

HBsAg clearance at the end of treatment indicated successful NA cessation in this study. Higher levels of HBsAg indicated increased risk of virologic relapse. Similar results were reported in some studies [21–23]. In contrast, Seto et al. reported that HBsAg levels at the beginning of entecavir treatment, entecavir cessation, and the subsequent rate of HBsAg reduction were not associated with virologic relapse [24]. The reason for these two opposite opinions is still unclear. HBsAg, which is downstream product of HBV cccDNA [25–27], may reflect levels of HBV cccDNA [28]. Higher levels of both hepatitis B surface and core-related antigens at the time of NA discontinuation were associated with relapse [28]. Lower end-of-treatment HBsAg levels may contribute to higher cumulative HBsAg loss rate after NA cessation [29]. A quite lengthy follow-up observation after NA cessation is necessary for evaluating the rate of HBsAg loss. A randomized controlled study from Berg T [30] found that HBsAg levels decreased in the 144 weeks follow-up after TDF therapy stopped. Study from Hadziyannis et al. [31] showed 39% of patients lose HBsAg in the 6-year follow-up after ADV therapy cessation. But it is assumed that NA therapy requires 52.2 years in order to achieve HBsAg clearance and that a finite treatment duration is unlikely [32]. As HBsAg clearance is not easy to achieve, other predictive factors should be investigated in the clinical practice.

In this study, age was a predictive factor for virologic relapse. Some studies have shown that age > 40 years predicted relapse in origin HBeAg positive patients [9, 23, 33]. Age served as a predictor for virologic relapse in origin HBeAg negative patients in other studies [21, 23]. We found no other predictive factors for virologic relapse, such as NA treatment duration or duration of negative HBV DNA maintenance. Chaung et al. [10] reported that HBeAg seroconversion and consolidation duration were not predictors for relapse. Findings from Chen et al. suggest that there were no predictive factors for relapse [34]. It seems that prolonged NA treatment or prolonged consolidation treatment cannot reduce the virologic relapse rate, which is not in accordance with the new updates of guidelines, comparing to the old ones, from APASL [35], suggesting more than 3 years of consolidation treatment in origin HBeAg positive patients to stop NA, or from EASL [36], suggesting more than 3 years of virological suppression in origin HBeAg negative patients to stop NA. But a systematic review [37] suggests that on-therapy virological remission (VR)  >  24 months offers higher chances of off-NA VR in patients with HBeAg negative chronic hepatitis B.

As mentioned above, there are difficulties to stop NA therapy without relapse. Discontinuation of NA therapy in CHB continues to be a hot topic with contrasting views in the recent liver meeting: patients may benefit from NA therapy cessation [38]; however there is no robust evidence to support treatment discontinuation [39].

This study has some limitations. First, the case number was small, since we recruited patients from only one center. Second, pretreatment HBV genotype results were not carried out, since diagnostic reagents were scarce and HBV genotype was not a routine test in the past ten years in China which is still a developing country. Third, further study on HBV-specific cytotoxic T lymphocytes immune response was not carried out.

## 5. Conclusion

NA cessation is safe under supervision. Increased vigilance was required in the first 24 weeks in origin HBeAg positive patients and the first 12 weeks in negative patients. Age, HBsAg level before NA cessation, and origin HBeAg status before NA treatment can be predictive factors for virologic relapse. HBV DNA can be predictive factor for retreatment.

## Figures and Tables

**Table 1:** Baseline characteristics of 62 patients before NA treatment cessation.

	origin HBeAg positive (*n* = 39)	origin HBeAg negative (*n* = 23)	All (*n* = 62)
Age, year	33.18 ± 8.33	44.39 ± 8.99	37.34 ± 10.11
Gender, male (%)	26 (66.7)	19 (82.6)	45 (72.6)
BMI, kg/cm^2^	21.31 ± 2.91	23.01 ± 3.12	21.95 ± 3.08
NA treatment, ratio of ETV : LDT : LAM : ADV : LAM + ADV^*∗*^	10 : 17 : 3 : 5 : 4	9 : 6 : 2 : 2 : 4	19 : 23 : 5 : 7 : 8
Duration of NA treatment, year	4.64 ± 2.63	4.33 ± 1.74	4.52 ± 2.33
Duration of negative HBV DNA maintenance, year	3.83 ± 2.15	3.68 ± 1.31	3.78 ± 1.87
Duration of HBeAg seroconversion maintenance, year	3.19 ± 2.47	-	-
EOT HBsAg, log⁡10(IU/mL)^#^	2.65 ± 1.22	2.36 ± 1.38	2.54 ± 1.28

^*∗*^NA: nucleos(t)ide analogues; ETV: entecavir; LDT: telbivudine; LAM: lamivudine; ADV: adefovir dipivoxil; ^#^EOT: end of treatment.

**Table 2:** Independent variables associated with virologic relapse in GEE models.

Parameters	Univariate		Multivariate	
	OR (95% CI)	*p*	OR (95% CI)	*p*
Sex				
Male	Ref	-	Ref	-
Female	1.12 (0.51–2.49)	0.774	1.14 (0.59–2.19)	0.704
Age, year	1.05 (1.01–1.08)	0.009	1.06 (1.02–1.10)	0.003
BMI, kg/m^2^	0.97 (0.87–1.09)	0.617	0.85 (0.75–0.95)	0.006
Duration of NA treatment, year	0.89 (0.74–1.08)	0.255	1.40 (1.14–1.73)	0.001
Duration of negative HBV DNA maintenance, year	0.81 (0.65–1.01)	0.066	0.52 (0.33–0.83)	0.005
HBsAg, log⁡10(IU/mL)	1.79 (1.32–2.45)	<0.001	2.21 (1.47–3.32)	<0.001
Origin HBeAg				
Negative	Ref	-	Ref	-
Positive	0.38 (0.19–0.79)	0.010	0.32 (0.14–0.74)	0.008

**Table 3:** Independent variables associated with retreatment in GEE models.

Parameters	Univariate		Multivariate	
	OR (95% CI)	*p*	OR (95% CI)	*p*
Sex				
Male	Ref	-	Ref	-
Female	1.75 (0.42–7.35)	0.445	2.17 (0.45–10.43)	0.332
Age, year	1.06 (0.98–1.14)	0.177	1.04 (0.92–1.18)	0.490
BMI, kg/m^2^	1.00 (0.80–1.26)	0.975	1.02 (0.78–1.34)	0.878
Duration of NA treatment, year	1.25 (0.81–1.95)	0.314	1.08 (0.53–2.20)	0.830
Duration of negative HBV DNA maintenance, year	1.45 (0.90–2.33)	0.130	1.11 (0.42–2.91)	0.830
HBV DNA, log⁡10(IU/mL)	1.28 (1.08–1.50)	0.003	1.34 (1.13–1.58)	<0.001
HBsAg, log⁡10(IU/mL)	0.69 (0.26–1.83)	0.460	0.64 (0.24–1.72)	0.382
Origin HBeAg				
Negative	Ref	-	Ref	-
Positive	1.07 (0.30–3.80)	0.921	1.23 (0.17–8.93)	0.838

## Abbreviations

NA: Nucleos(t)ide analogues
HBV: Hepatitis B virus
CHB: Chronic hepatitis B
HCC: Hepatocellular carcinoma
ETV: Entecavir
LDT: Telbivudine
LAM: Lamivudine
ADV: Adefovir dipivoxil
TDF: Tenofovir disoproxil fumarate
HBsAg: Hepatitis B surface antigen
HBeAg: Hepatitis B e antigen
AST: Aspartate transaminase
ALT: Alanine transaminase
CD4^+^T: CD4 positive T lymphocytes
CD8^+^T: CD8 positive T lymphocytes
Th1: Type 1 helper T lymphocytes
Th2: Type 2 helper T lymphocytes
ULN: Upper limit of normal
EOT: End of treatment
VR: Virological remission.
